# Assessing character strengths in hungary: exploring factor structure and reliability of VIA-IS-M

**DOI:** 10.1186/s40359-025-03141-w

**Published:** 2025-07-23

**Authors:** Adrienn Molnár, Győző Kurucz, Szilvia Fodor

**Affiliations:** 1 Institute of Psychology, Faculty of Humanities, University of Debrecen, Egyetem sugárút 1, Debrecen, 4032 Hungary; 2 Institute of Psychology, Faculty of Education and Psychology, ELTE Eötvös Loránd University, Izabella u. 46, Budapest, 1064 Hungary

**Keywords:** Positive psychology, Character strengths, Measurement of character strengths, VIA-IS-M

## Abstract

**Background:**

Character strengths are a prevalent topic of positive psychology research. Recently, the questionnaires suitable for measuring the 24 character strengths (VIA-IS) have been revised. This new version, called VIA-IS-R, has a briefer form, the 96-item VIA-IS-M. Despite the widespread usage and measurement of the character strengths, the revised version of the questionnaire has yet to be utilised in Hungary. Therefore, the study aimed to test and validate the VIA-IS-M questionnaire in the Hungarian context.

**Methods:**

We conducted four online studies on different adult samples, with 1878 participants in total. In addition to character strengths, satisfaction with life, well-being, burnout, and mental health were measured to analyse criterion validity.

**Results:**

In Study 1, the internal consistency of the scales was low in some cases (Gratitude, Judgment, Kindness). After modification, reliabilities were acceptable in the following studies. Weak-to-moderate correlations were observed between character strengths and criterion variables, consistent with prior expectations. Character strengths were used as predictor variables in the regression models, and they demonstrated sufficient predictive power for all the criterion variables in all studies (R^2^ = 0.43-71). According to exploratory factor analysis, 3-factor solutions were suitable for the data and aligned with recent studies on character strengths’ factor structure.

**Conclusions:**

Overall, the VIA-IS-M-H demonstrates acceptable reliability and validity levels and is suitable for assessing character strengths in the Hungarian context.

## Introduction

The Classification of Character Strengths and Virtues, developed by Peterson and Seligman [1], is one of the most comprehensive models for assessing character. Character strengths, by definition, are positively valued traits expected to contribute to good life. According to Niemiec [2], character strengths reflect our basic identity, promote positive outcomes and contribute to collective good. The classification identifies six core virtues (Courage, Justice, Humanity, Temperance, Wisdom, and Transcendence) and encompasses 24 character strengths, each aligned with one of these core virtues. All individuals possess a unique combination of the 24 character strengths. Three to seven strengths can be part of an individual’s core identity, the so-called signature strengths [1]. Signature strengths are essential to an individual, as they are effortlessly expressed, energising, and provide a profound sense of fulfilment and enthusiasm [3]. Character strengths have been widely studied and applied in various contexts, including education [4], workplace and organisations [5, 6], psychotherapy [7], strength-based psychological interventions [8], and with diverse populations, such as individuals with disabilities [9] and older adults [10].

To assess the 24 character strengths in the adult population, a self-report questionnaire, the VIA Inventory of Strengths (VIA-IS) [11], was developed, consisting of 240 positively keyed items, with 10 items measuring each strength. The VIA-IS was utilised in numerous studies and has been adapted to multiple languages, including German [12] Spanish [13], Hindi [14], and Japanese [15], enabling its use in a global and cross-cultural context. Additionally, two shorter versions of the questionnaire are available: the VIA-120, which consists of 120 items [1, 16] (5 items per strength), and the VIA-72 [1], which consists of 72 items (3 items per strength).

Empirical studies investigating the factor structure of the VIA Inventory of Strengths (VIA-IS) have yielded varied results, with some studies proposing four-factor solutions [17–20] and others suggesting five-factor solutions [12, 21–24]. However, the number of factors identified ranges from one to six, indicating a lack of consistent factor structure for the VIA-IS instrument [25].

**Fig. 1:**
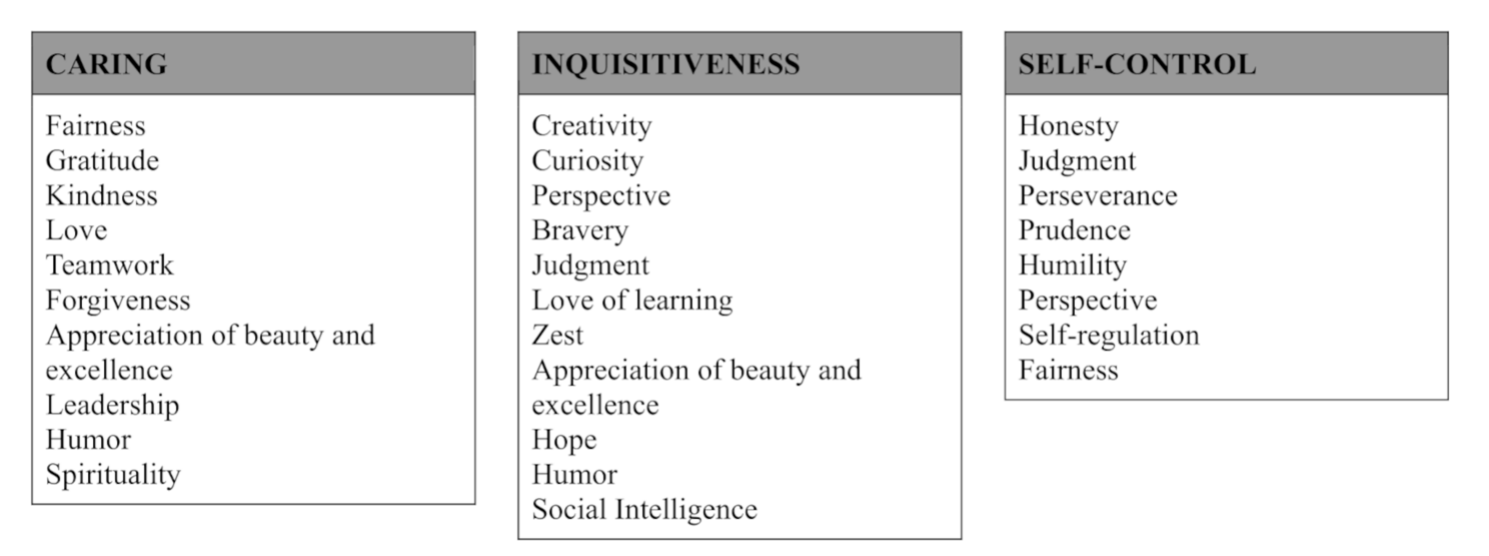
Empirically supported factor structure suggested by McGrath [26]

McGrath [26] proposed a three-factor solution, which has shown stability across different samples. These factors were called Caring, Inquisitiveness, and Self-Control (see Fig. 1). Although this three-factor model is not a perfect fit, five character strengths (Appreciation of Beauty and Excellence, Fairness, Humor, Judgment, and Perspective) have cross-loadings on two factors [27]. More recently, Partsch et al. [28] also suggested a three-factor model, which differs from McGrath’s [24] solution. It is worth noting that Partsch et al. [28] utilised the IPIP-VIA-R instruments, so the results are different from those of McGrath [26], who used the VIA-IS.

While the VIA Inventory of Strengths (VIA-IS) is widely used with adequate psychometric properties [12], McGrath [29] identified several limitations of the measurement. First, the exclusive use of positively keyed items might cause a response bias to result in a very high score. He highlighted further issues that need to be addressed, such as the lack of transparency in item development, the excessive length, the absence of virtue-level scales, and the heterogeneous content within scales that complicate interpretation and construct clarity.

Recognising limitations in the original VIA Inventory of Strengths (VIA-IS), the VIA Institute undertook an extensive revision process to create a shorter assessment tool that includes negatively keyed items and items of varying difficulty and has a rigorously documented development process [29]. The revision process involved reviewing existing items and developing new ones, focusing on incorporating reverse-keyed items for each scale. The outcome of the extensive revision is the VIA Inventory of Strengths-Revised (VIA-IS-R), comprising 192 items with eight items measuring each character strength [29]. Additionally, two shorter forms were developed:

- VIA-IS-P (“Positive”): Uses only positively keyed items, which is ideal for respondents who may struggle with negatively keyed items (96 items).
- VIA-IS-M (“Mixed”): Includes two positively and two negatively keyed items per character strengths (96 items) (except for Teamwork, which has one negatively and three positively keyed items), addressing response biases inherent in positively formulated items [30].

The VIA Inventory of Strengths-Revised (VIA-IS-R) demonstrated satisfactory reliability and validity in initial validation by McGrath and Wallace [30], showing strong convergence with the VIA-120. Further proof by Vylobkova et al. [31] in a German sample found higher internal consistency for the VIA-IS-R compared to the original VIA-IS, with both instruments displaying similar correlations with thriving and moral behaviours. The shorter forms, the VIA-IS-P and VIA-IS-M, also indicated acceptable internal consistency [30, 31].

VIA-IS-R offers significant advantages, including a shorter length and a more rigorous item selection process, considering comprehensive criteria to ensure the quality and relevance of the included items. Additionally, the development process of the VIA-IS-R is transparent and well-documented, enhancing its credibility and facilitating replication and further research. Overall, empirical evidence supports the psychometric adequacy and validity of the VIA-IS-R, indicating its suitability for assessing the 24 character strengths [30–33].

### Overview of studies

The VIA-IS-R and its shorter versions, including the VIA-IS-M, have demonstrated suitability for measuring character strengths [30, 31]. Our project aimed to adapt the VIA-IS-M to the Hungarian language, assess its internal consistency and criterion validity, and examine the factor structure of character strengths, which were carried out through four separate studies. While character strengths research is well-established globally, the revised VIA instruments have not yet been utilised in Hungary. Thus, this study marks a significant step toward advancing character strengths research within the Hungarian context.

Considering the revised versions of the VIA questionnaires, we selected the VIA-IS-M for validation. The shorter length of VIA-IS-M (96 items compared to the 192 items in VIA-IS-R) is more practical and reduces the respondent’s burdens. Additionally, including negatively keyed items in the VIA-IS-M addresses potential ceiling effects commonly observed in the VIA-IS [29], distinguishing it from the VIA-IS-P, which only includes positively keyed items. McGrath et al. [31] highlighted the VIA-IS-M’s suitability for research purposes, further supporting its selection for this study.

The validation process was conducted through four studies with distinct objectives, methodologies, and statistical analyses (see Table 1).

**Table 1:** Overview of the four studies, the measures used in them, and the statistical analyses carried out

	Study 1	Study 2	Study 3	Study 4
**Sample size**	*N* = 1001	*N* = 262	*N* = 223	*N* = 392
**Measures**	- VIA-IS-M-H^a^ - PERMA-Profiler^d^ - SWLS-H	- VIA-IS-M-H^b^	- VIA-IS-M-H^c^ - Workplace PERMA questionnaire	- VIS-IS-M-H^c^ - PERMA-Profiler^e^ - SWLS-H - MET
**Statistical analyses**	- Descriptive analysis - Reliability - Criterion validity	- Descriptive analysis - Item selection - Reliability - Exploratory factor analysis	- Descriptive analysis - Reliability - Criterion validity - Exploratory factor analysis	- Descriptive analysis - Reliability - Criterion validity - Exploratory factor analysis

*Notes.*^a^ provisional Hungarian translation of the VIA-IS-M questionnaire; ^b^ modified and expanded version of the VIA-IS-M-H; ^c^ suggested final version of the VIA-IS-M-H; ^d^ unofficial Hungarian translation of the PERMA-Profiler questionnaire; ^e^ official translation of the PERMA-Profiler questionnaire

In the first study, we tested the provisional Hungarian translation of the VIA-IS-M questionnaire on a larger sample to examine the reliability of the scales and criterion validity through the study of the relationships between the character strengths and certain criterion variables (well-being and satisfaction with life). We also aimed to determine to what extent the character strengths could predict the criterion variables.

Based on the findings from the first study, in which some scales exhibited inadequate reliability, we developed a modified version of the VIA-IS-M-H questionnaire that includes additional items for the second study. Our goal was to create a final version of the questionnaire that accurately measures character strengths with sufficient reliability. We completed the item selection to propose this final version of the VIA-IS-M-H. Additionally, we examined the higher-level organization of the character strengths assessed by this final version through exploratory factor analysis.

In the third study, we examined the reliability and criterion validity of the suggested final version of the VIA-IS-M-H questionnaire among a specific population– primary and secondary school teachers. To examine criterion validity, we investigated the correlations between strengths and criterion variables (workplace well-being and burnout) and verified their predictability. Similarly, to the second study, we explored the higher-level organisation of character strengths through exploratory factor analysis.

The fourth study, which aimed to be similar to the third study, involved a sample drawn from the general population. Although the findings of the third study indicated satisfactory psychometric properties, we wanted to assess the functionality of the proposed final version of the questionnaire on a broader sample. This study used different criterion variables—such as general well-being, life satisfaction, and mental health—to examine criterion validity. Similarly to the previous two studies, we explored the higher-level organisation of character strengths through exploratory factor analysis.

We also conducted a cross-study analysis to examine the consistency between the factor solutions found in the second, third, and fourth studies. This analysis provided an opportunity to assess the stability of the potential factor structure underlying the character strengths measured by the VIA-IS-M-H across different samples. Analysing the factor structure also helps us to judge if it is congruent with the empirically derived factor structure suggested by McGrath [26].

### Measures

#### Hungarian translation of the VIA-IS-M questionnaire (VIA-IS-M-H)

The VIA-IS-M is a 96-item questionnaire that measures the 24 VIA character strengths using 4 items each [29]. The scales consist of two positive and two negative items (except for Teamwork, which includes only one negative item). The respondents had to evaluate the items on a 5-point Likert scale, where 1 indicated “not like me at all” and 5 indicated “very much like me.”

The VIA Institute permitted and supervised the initial translation of the questionnaire. Two independent translators translated the questionnaire’s items into Hungarian, and after reconciling the translations, they were back-translated into English to verify content equivalence.

Throughout the studies, we worked with three different versions of the questionnaire. The first version was the provisional translation used in the first study. The second one, a modified and expanded version, was used in the second study. The third version, the suggested final version was used in the third and fourth studies.

In the second study, certain items of the VIA-IS-M-H were retranslated to reduce the possibility of potential misinterpretations and improve the reliability of the identified problematic scales. A total of 7 item translations were revised, affecting 6 character strengths. Additionally, new items were translated from the items of the VIA-IS-R questionnaire, expanding the questionnaire with the hope of obtaining scales with better reliability during item selection, especially for those strengths that showed weak reliability in the first study. A total of 23 new items were added to the questionnaire, involving 14 character strengths. The modified version of the questionnaire thus created contained 119 items, with varying numbers of items ranging from 4 to 7 for each character strength.

Based on the results of the second study, the suggested final version of the questionnaire was developed. It consists of 96 items, with 4 items per scale, including two positive and two negative items, except for Teamwork, which had only 1 negative item, and Perseverance, which had 3 negative items. No further modifications were made to the questionnaire in the third and fourth studies.

### Hungarian adaptations of the PERMA-Profiler

The PERMA-Profiler questionnaire [34] was used to measure well-being. The questionnaire consists of 23 items, of which 15 measure the five dimensions of well-being based on Seligman’s [35] model: Positive Emotions, Engagement, Relationships, Meaning, and Accomplishments. The remaining 8 items measure negative emotions (3 items), physical health (3 items) and loneliness (1 item). Respondents had to rate themselves on an 11-point Likert scale for each item, where 0 represented “never”/“ not at all” and 10 represented “always”/“ completely” dependent on item content.

In the first study, we worked with an unvalidated questionnaire version. During the translation process, two independent translators translated the items, and after reconciling the translations, we verified their content equivalence with the original items. At the time of the first study, we were unaware of the ongoing official adaptation of the Hungarian PERMA-Profiler questionnaire. Nevertheless, we used the adopted and validated questionnaire by Varga et al. [36] in the fourth study.

### Workplace PERMA questionnaire

Kun et al. [37] developed the Workplace PERMA questionnaire based on Seligman’s (2011) PERMA model. The questionnaire consists of a total of 35 items, which measure the five dimensions of the PERMA model: Positive emotions (5 items), Engagement (6 items), Relationships (5 items), Meaning (6 items), Accomplishments (5 items), as well as an additional dimension the Negative aspects of work (8 items). Respondents must rate themselves on a 5-point Likert scale for each item, where 1 indicates “strongly disagree”, and 5 indicates “strongly agree”.

### Hungarian adaptation of the satisfaction with life scale (SWLS-H)

We used the Hungarian version of the Satisfaction with Life Scale (SWLS-H, Martos et al. [38]). The assesses an individual’s subjective evaluation of life satisfaction through 5 positively worded items. Responses can be given on a 7-point Likert scale, where 1 indicates „strongly disagree” and 7 indicates „strongly agree”.

### Mental health test (MET)

The questionnaire measuring mental health was developed by Vargha et al. [39]. The questionnaire consists of 17 items, of which 3 items measure Well-being, 3 items measure Savoring, 5 items measure Creative and executive efficiency, 3 items measure Self-regulation, and 3 items measure Psychological resilience. Respondents must respond to each item on a 6-point Likert scale, where 1 indicates „not at all characteristic” and 6 indicates „completely characteristic”.

### Statistical analyses

We used the R 4.1.2 statistical programming language (R Core Team [40]) for data analysis, and we utilised additional packages for performing specific analyses.

### Filtering out inconsistent responses

We excluded participants from the statistical analysis who had inconsistent responses to mitigate the impact of insufficient effort in responding. These inconsistent responses could stem from not carefully reading the questions, not paying sufficient attention and effort to the response process, or simply providing random responses.

To identify inconsistent responses, we used item pairs in the VIA-IS-M-H questionnaire that consisted of one positive and one negatively worded item, directly contradicting each other (e.g. „I always keep my promises” and „I often break my promises”). In total, we found seven such item pairs in the questionnaire. A participant was considered an insufficient effort responder and excluded from the analysis if they either strongly agreed or disagreed with both items in at least one of these item pairs. Since these item pairs remained unchanged in all versions of the VIA-IS-M-H, we used the same pairs for all studies.

### Descriptive analysis

During the descriptive statistical analysis of the character strengths measured with the VIA-IS-M-H, we calculated the mean and standard deviation of the strengths. We also calculated the skewness of their empirical distribution to assess potential ceiling or floor effects.

### Reliability and item-selection

The reliability of the measurement scales used in the studies was assessed using the Cronbach’s α coefficient, which measures the internal consistency of the scales. As a reliability measure, values of α higher than or equal to 0.6 were considered acceptable, values higher than or equal to 0.9 were considered excellent, and values lower than 0.6 were considered weak.

In the second study, the item selection for each scale was based on item-rest correlations and the expected Cronbach’s α values when excluding individual items. An item was considered problematic if its item-rest correlation significantly deviated from the rest of the items in the scale and/or if its removal would result in an α value exceeding the upper limit of the 95% confidence interval calculated for the entire scale, as suggested by Feldt et al. [41] For the analyses, we used the „alpha” function from the „psych” package [42].

### Validity

To assess the VIA-IS-M-H questionnaire’s criterion validity, we first examined the linear relationships between character strengths and criterion variables using Pearson’s correlation coefficient. Life satisfaction and well-being were selected as the primary criterion variables, as prior studies have consistently demonstrated a strong positive relationship between character strengths and these constructs [22, 43, 44].

We conducted multiple linear regression analyses to determine the extent to which character strengths are suitable for predicting criterion variables. We used character strengths as predictor variables in the regression models and controlled for age and gender (dummy-coded). We calculated non-standardised model parameters and computed the coefficient of determination adjusted for the number of independent variables to measure the model’s explanatory power. Considering the expected linear relationships among character strengths, we checked for the level of multicollinearity in the models using the variance inflation factor (VIF) calculated for each independent variable. VIF values above 5 were considered problematic, indicating strong multicollinearity. To calculate VIF, we used the “car” package [45].

For measuring the criterion variables, which consisted of multiple subscales, we performed principal component analysis to create a composite indicator. In this process, we used the score of the first principal component as the measure for the criterion variable.

### Exploratory factor analysis

We examined the higher-order organisation of character strengths using exploratory factor analysis. We examined the latent structure at the character strength level rather than the item level. Before analysis answers to items were aggregated to yield measures of character strengths. To assess the suitability of the data for factor analysis, we calculated the Kaiser-Meyer-Olkin measure of sampling adequacy (MSA). We considered values higher than or equal to 0.8 to be acceptable.

We conducted a parallel analysis to determine the ideal number of factors and employed Velicer’s maximum average partial (MAP) procedure. We aimed to explore similar factor structures across different samples and compare them to the three-factor structure of character strengths—the empirically derived virtues model—proposed by McGrath [27]. The suggested number of factors based on the aforementioned procedures were considered for informational purposes only, and in each study, we fitted a three-factor solution to the data.

To extract factor loadings, we used principal axis factoring with Promax rotation (with a power of 4) to allow for correlations between the obtained factors.

Within the “psych” package [42], we used the “fa.parallel” function for parallel analysis, the “vss” function for running the MAP procedure, and the “fa” function for conducting exploratory factor analysis.

### Similarity of factor solutions

We compared the factor solutions obtained in the different studies to determine whether the character strengths were grouped into similar factors across different samples, indicating the stability of the obtained factor solution. For this purpose, we used the commonly employed Rand and adjusted Rand indices to measure the agreement between different clustering solutions. Also, we used Tucker’s φ to assess the level of congruence between factor solutions in our studies. Together, these two indices can be used to assess the similarity between factor solutions on a coarser, structural level as well as on a finer level, based on the similarity of factor loadings.

As a first step in assessing correspondence, we assigned each character strength to a specific factor within each factor solution. We unambiguously assigned each strength to a single factor based on the highest factor loading. Subsequently, we calculated the Rand and adjusted Rand index for each pair of factor solutions of Study 2 and Study 3; Study 2 and Study 4; and Study 3 and Study 4 using the “rand.index” and “adj.rand.index” functions from the “fossil” package [46].

The Rand index measures the similarity between two factor solutions by considering all possible pairwise combinations of character strengths. It determines how many pairs of strengths are assigned to the same or different factors across both solutions. The adjusted Rand index further corrects for the expected level of random agreement. The Rand index ranges from 0 to 1 (the adjusted Rand index can go below 0), where 1 indicates perfect agreement between the two factor solutions, 0 indicates complete lack of agreement (Rand index), and it corresponds to the level of chance agreement for the adjusted Rand index.

We are not aware of the Rand index being used in a similar context or of the guidelines for interpreting its values in such a context. Therefore, we cannot interpret when the agreement is considered high or acceptable. However, we considered values of Rand indices closer to 1 to indicate a higher level of agreement and values of adjusted Rand indices above 0 and closer to 1 to indicate a higher than chance agreement.

As a second step, we used Tucker’s φ [47], which is calculated as a cosine of vectors of factor loadings from two different factor solutions. The φ index measures congruence on the level of factor loadings, where the maximum value of the index can be obtained if the factor loadings in the two factor solutions differ only by a scaler. A recent recommendation based on factor analysis experts’ opinions compiled by Lorenzo-Seva & ten Berge [48] was used to interpret the level of congruence based on φ. According to this recommendation, values 0.95 and above refer to good congruence, values between 0.85 and 0.94 to fair congruence, and values below 0.85 to the lack of congruence.

We calculated Tucker’s φ for each factor separately for each pair of studies using the “factor.congruence” function from the “psych” package [40].

Our goal using exploratory factor analysis was to assess the stability of the latent structure across different samples and its congruence with McGrath’s [26] proposed classification of character strengths. As this classification is rather a summarisation of the results of previous factor analytic studies without hard limits between categories (i.e. several strengths are included in multiple categories, see Fig. 1), congruence was only judged on a qualitative level rather than strictly examining the fit of some specific factor structure.

## Study 1

### Method

#### Participants

A total of 1221 individuals participated in the study. We used convenience sampling and recruited participants through social media platforms. Participants took part in the study voluntarily, and no compensation was provided. The inclusion criteria for participation were being at least 18 years old. One participant was excluded from the analysis due to being under 18 years old, and an additional 219 participants were excluded due to suspected inconsistent responses during the survey. Thus, our final sample consisted of 1001 respondents. The average age was 38.5 years (SD = 14.05, range: 18–76 years). The majority of participants were female (85%, *n* = 851), in a relationship (65.9%, *n* = 660), and had a higher education degree or were pursuing higher education studies (68.2%, *n* = 683).

### Procedure

The study was approved by the United Ethical Review Committee for Research in Psychology (reference number 2020/127). We published our questionnaire on Google Forms, which participants could access through the link shared on social media platforms.

The set of questionnaires included the provisional Hungarian translation of the VIA-IS-M questionnaire (VIA-IS-M-H), the PERMA-Profiler questionnaire translated by the authors, the SWLS-H questionnaire, and basic demographic questions (gender, age, highest level of education, relationship status).

### Results

#### Descriptive analysis

Among the character strengths, we found the highest average scores for Love of Learning (M = 4.39), Curiosity (M = 4.37), and Honesty (M = 4.33) - for these, the skewness indices indicate a significant shift towards higher scores, suggesting a slight ceiling effect. The lowest average score was found for Humility (M = 2.97) (see Table 2.).

**Table 2:** Descriptive statistics from study 1 (incl. Cronbach’s α for strengths, and pearson correlation coefficients with criterion variables)

Strength	M (SD)	Skewness	α	*P*	E	*R*	M	A	Well-being	SWLS-H
Creativity	3.81 (0.80)	-0.56	0.78	0.32^***^	0.37^***^	0.16^***^	0.39^***^	0.38^***^	0.37^***^	0.24^***^
Curiosity	4.37 (0.66)	-1.49	0.80^a^	0.36^***^	0.51^***^	0.19^***^	0.38^***^	0.40^***^	0.41^***^	0.27^***^
Judgment	3.43 (0.70)	-0.11	0.59	0.14^***^	0.07	0.07	0.18^***^	0.20^***^	0.17^***^	0.10
Love of learning	4.39 (0.75)	-1.45	0.88^a^	0.27^***^	0.47^***^	0.13^**^	0.33^***^	0.36^***^	0.34^***^	0.23^***^
Perspective	3.79 (0.79)	-0.53	0.81^a^	0.30^***^	0.22^***^	0.20^***^	0.41^***^	0.42^***^	0.38^***^	0.26^***^
Bravery	3.60 (0.83)	-0.35	0.77	0.38^***^	0.26^***^	0.21^***^	0.45^***^	0.47^***^	0.43^***^	0.26^***^
Perseverance	3.38 (0.89)	-0.48	0.76	0.40^***^	0.22^***^	0.24^***^	0.47^***^	0.60^***^	0.48^***^	0.34^***^
Honesty	4.33 (0.65)	-1.17	0.79^a^	0.26^***^	0.18^***^	0.16^***^	0.34^***^	0.41^***^	0.33^***^	0.19^***^
Zest	3.21 (0.99)	-0.38	0.82	0.67^***^	0.32^***^	0.40^***^	0.59^***^	0.54^***^	0.64^***^	0.48^***^
Love	3.76 (1.02)	-0.54	0.87	0.51^***^	0.22^***^	0.48^***^	0.46^***^	0.41^***^	0.53^***^	0.37^***^
Kindness	4.04 (0.71)	-0.78	0.66	0.29^***^	0.21^***^	0.28^***^	0.30^***^	0.28^***^	0.33^***^	0.19^***^
Social intelligence	3.86 (0.82)	-0.83	0.78	0.36^***^	0.20^***^	0.28^***^	0.40^***^	0.40^***^	0.41^***^	0.26^***^
Teamwork	3.59 (0.88)	-0.43	0.78^a^	0.36^***^	0.20^***^	0.31^***^	0.32^***^	0.26^***^	0.37^***^	0.23^***^
Fairness	3.71 (0.85)	-0.56	0.81	0.23^***^	0.12^**^	0.18^***^	0.23^***^	0.17^***^	0.24^***^	0.15^***^
Leadership	3.35 (1.00)	-0.31	0.80^a^	0.37^***^	0.23^***^	0.25^***^	0.41^***^	0.43^***^	0.42^***^	0.27^***^
Forgiveness	3.17 (0.89)	-0.06	0.72	0.38^***^	0.12^**^	0.29^***^	0.30^***^	0.23^***^	0.34^***^	0.30^***^
Humility	2.97 (0.72)	-0.19	0.54	− 0.12^**^	− 0.02	− 0.09	− 0.12^**^	− 0.13^**^	− 0.13^**^	− 0.09
Prudence	3.52 (0.81)	-0.34	0.75	0.21^***^	0.11^*^	0.15^***^	0.30^***^	0.34^***^	0.28^***^	0.20^***^
Self-regulation	3.45 (0.75)	-0.47	0.65	0.27^***^	0.13^**^	0.16^***^	0.36^***^	0.41^***^	0.34^***^	0.22^***^
Appreciation of beauty	3.96 (0.78)	-0.58	0.63^a^	0.21^***^	0.33^***^	0.15^***^	0.21^***^	0.20^***^	0.24^***^	0.18^***^
Gratitude	3.74 (0.68)	-0.52	0.53^a^	0.56^***^	0.33^***^	0.43^***^	0.49^***^	0.41^***^	0.55^***^	0.42^***^
Hope	3.93 (0.89)	-0.89	0.80	0.77^***^	0.32^***^	0.51^***^	0.74^***^	0.69^***^	0.78^***^	0.61^***^
Humor	3.49 (0.94)	-0.45	0.78	0.41^***^	0.17^***^	0.25^***^	0.33^***^	0.30^***^	0.37^***^	0.27^***^
Spirituality	3.26 (1.19)	-0.30	0.85^a^	0.21^***^	0.15^***^	0.15^***^	0.23^***^	0.20^***^	0.23^***^	0.14^***^

*Notes*. ^*^p <.05; ^**^p <.01; ^***^p <.001; ^a^ improvable through item-selection

### Reliability

For the PERMA-Profiler scales, Cronbach’s α values ranged from 0.79 to 0.91, indicating acceptable to excellent reliability, except for the Engagement scale, which had an α of 0.50, indicating poor reliability. Regarding the SWLS-H, α was 0.88, indicating adequate reliability.

The results for the VIA-IS-M scales were mixed. Most scales showed Cronbach’s α values above 0.6, indicating an acceptable to excellent level of reliability. However, specific scales had lower reliability (Judgment, Humility, Gratitude). Additionally, for several scales, it was found that the reliability would significantly improve if items were omitted (see Table 2).

### Validity

To examine validity, in addition to exploring the correlates of well-being subscales, we created a simple measure of well-being based on the five main scales of the PERMA-Profiler using principal component analysis. The first principal component obtained from the analysis explained approximately 70.5% of the variance of the original scales. The loadings of the Positive emotions, Engagement, Relationship, Meaning, and Accomplishments scales were 0.52, 0.21, 0.47, 0.56, and 0.40, respectively. Therefore, higher scores on this first principal component indicated a higher level of well-being.

The character strengths generally showed moderate to strong correlations with well-being and life satisfaction as measured by the SWLS-H (see Table 2.). Additionally, we examined the relationships between character strengths and the PERMA dimensions. The indicated show that — with the exception of Humility and Judgement — all character strengths were positively related to all PERMA dimensions, with effect sizes ranging from small to large.

Based on the multiple linear regression analysis results, the character strengths significantly explained the variance in well-being and life satisfaction, with the models explaining 71% and 43% of the variance. In both models, the strengths of Hope, Gratitude, Zest, Love, Prudence, Perseverance, and Fairness had significant effects. In contrast, Bravery, Forgiveness, and Spirituality only had significant effects on life satisfaction, and Humility only significantly affected well-being. It is worth noting that the effects of Bravery, Fairness, Humility, and Spirituality on the outcome variable were negative (see Table 3.).

Multicollinearity in the models was moderate, with variance inflation factors ranging from 1.15 to 2.75 for the character strengths.

**Table 3:** Results of multiple linear regression analyses across three of the studies

	Dependent variable					
	Study 1		Study 3		Study 4	
Model parameters	Well-being	SWLS-H	Well-being	Well-being	SWLS-H	Mental health
**Intercept**	-15.83^***^	1.14^**^	-5.28^***^	-12.34^***^	0.94	-8.83^***^
**VIA strengths**						
Creativity	0.08	0.02	-0.14	-0.33^*^	-0.20^*^	-0.09
Curiosity	0.25	0.06	0.17	0.07	0.24^*^	0.26^*^
Judgment	-0.09	-0.11	-0.05	-0.03	0.11	0.05
Love of learning	0.06	0.00	-0.04	0.20	-0.22^*^	-0.04
Perspective	0.04	0.05	-0.16	0.34	0.17	0.11
Bravery	0.02	-0.14^*^	-0.23^*^	0.09	0.12	0.23^*^
Perseverance	0.26^**^	0.11^*^	-0.04	-0.27	-0.08	-0.12
Honesty	0.02	-0.11	-0.26	0.24	0.12	0.00
Zest	0.57^***^	0.17^***^	0.52^***^	0.55^***^	0.18^*^	0.18^*^
Love	0.52^***^	0.12^**^	0.08	0.38^**^	0.09	0.13
Kindness	0.13	-0.01	0.10	-0.16	-0.14	-0.14
Social intelligence	0.04	-0.04	-0.07	-0.05	-0.07	0.15
Teamwork	0.07	-0.07	0.13	-0.03	0.02	0.02
Fairness	-0.29^***^	-0.10^*^	0.15	-0.17	-0.12	-0.02
Leadership	-0.02	0.02	0.09	0.17	0.13	0.00
Forgiveness	0.15	0.15^***^	0.23^**^	0.20	0.20^**^	0.26^***^
Humility	-0.20^*^	-0.05	-0.18	-0.13	-0.04	0.03
Prudence	0.34^**^	0.14^*^	0.23	-0.13	0.01	0.04
Self-regulation	0.08	0.00	0.04	0.40^*^	0.15	0.17
Appreciation of beauty	-0.01	0.01	0.21	0.00	-0.09	0.04
Gratitude	0.70^***^	0.27^***^	0.00	0.00	-0.04	-0.02
Hope	1.69^***^	0.65^***^	0.69^***^	1.87^***^	0.66^***^	0.84^***^
Humor	0.04	0.01	0.09	0.14	-0.01	0.35^***^
Spirituality	-0.09	-0.07^*^	-0.14^*^	0.04	0.11	-0.01
**Control variables**						
Gender	-0.16	0.09	0.15	0.54^*^	0.41^**^	0.12
Age	-0.02^***^	-0.01^*^	-0.01	-0.02^**^	-0.02^***^	0.00
*R* ^*2*^	0.71^***^	0.43^***^	0.59^***^	0.66^***^	0.46^***^	0.65^***^

Notes. * *p* <.05; ** *p* <.01; *** *p* <.001; gender was coded as male = 0, female = 1; non-standardised model parameters; R2 is the adjusted measure of determination

### Discussion

The first study aimed to test the internal consistency and criterion validity of the initial version of the VIA-IS-M-H questionnaire on a larger sample (*N* = 1001). The results indicated that the questionnaire demonstrated acceptable reliability for most scales. However, Gratitude (α = 0.53), Humility (α = 0.54) and Judgement (α = 0.59) scales displayed low reliability. Kindness (α = 0.66), Self-Regulation (α = 0.65), and Appreciation of Beauty and Excellence (α = 0.63) scales had low, yet acceptable, reliability scores. Additionally, the reliability of several scales could be improved by removing certain items (see Table 2).

Most character strengths showed robust positive relationships with well-being, while Humility and Judgement showed no or slight negative relation to the well-being dimension. These results align with the findings of Wagner et al. [43]. Regression models explained 71% of the variance in well-being and 43% in life satisfaction, with Hope, Gratitude, and Zest emerging as consistent predictors.

These findings underscore the necessity of modifying scales with lower reliability (e.g., Gratitude, Humility, and Judgement) to create a more reliable measurement, which will be addressed in Study 2.

## Study 2

### Method

#### Participants

A total of 312 individuals participated in the survey. Convenience sampling was employed, and participants were recruited through social media platforms. Participation was voluntary and anonymous, and no compensation was provided. The only requirement for participation was being 18 years or older.

Five participants were excluded from the analysis as they were under 18 years old, and an additional 45 participants were excluded due to potentially inconsistent responses. Thus, the final sample suitable for analysis consisted of 262 participants. Most participants were female (86.3%, *n* = 226) and had a higher education degree or were pursuing higher education studies (72.9%, *n* = 191). The average age in the sample was 35.7 years (SD = 13.28, range: 18–73 years).

### Procedure

The survey was administered using Google Forms, and participants could access it through links posted on social media.

The survey included a modified and expanded version of the VIA-IS-M-H, consisting of 119 items. In this version of the questionnaire, some specific scales consisted of 5 items (Curiosity, Courage, Perseverance, Honesty, Leadership, Appreciation of beauty and excellence). Some were expanded to 6 items (Judgement, Love of Learning, Kindness, Teamwork, Forgiveness, Self-Regulation, and Gratitude), and the Humility scale consisted of 7 items. In this expanded version a total of 7 items’ translation was modified including Curiosity (1), Judgment (1), Perseverance (1), Humility (1), Self-regulation (2), Creativity (1), and a total 23 new items were added for the strengths Curiosity (1), Judgment (2), Love of learning (2), Bravery (1), Perseverance (1), Honesty (1), Kindness (2), Appreciation of beauty (1), Gratitude (2), Teamwork (2), Leadership (1), Forgiveness (2), Humility (3), and Self-regulation (2).

Basic demographic questions (gender, age, highest level of education) were also measured.

### Results

#### Item selection and reliability

Based on the first study’s results, we revised the translation of all the items in the questionnaire. We modified those items that were particularly difficult to interpret in the Hungarian language and context (see Table 4). This study utilised an expanded version of the VIA-IS-M, incorporating additional items from the VIA-IS-R. The extra items were added to improve the subscales’ reliability of the previously identified problematic scales (e.g. Humility, Gratitude). After item selection- which was based on item-rest correlations and the expected Cronbach’s α values when excluding individual items- a total of 7 items were replaced with alternatives from the VIA-IS-R, involving 5 character strengths (see Table 4). The finalised scales comprised two positive and two negative items for each character strength. However, an exception was made for the Teamwork scale, which had only one negative item, and the Perseverance scale, which included 3 negative items. The proposed modifications were finalised with the approval of the VIA Institute. The reliability of the scales for each character strength proved to be acceptable (alpha > 0.6) (see Table 4).

### Descriptive analysis

Similar to the first study, we found the highest averages for Love of Learning (M = 4.56), Curiosity (M = 4.44), and Honesty (M = 4.29), although Kindness (M = 4.23) and Appreciation of Beauty (M = 4.10) scales also had relatively high average scores. The scores exhibit significant skewness for some character strengths (Love of Learning, Curiosity, Honesty), indicating a slight ceiling effect with a notable frequency of higher scores (see Table 4).

**Table 4:** Descriptive statistics of study 2 (incl. Cronbach’s α and modifications applied to individual strengths)

Strength	M (SD)	Skewness	Updates to items	α
Creativity	3.95 (0.78)	-0.60	1 translation modified (item 27)	0.82
Curiosity	4.44 (0.60)	-1.50	1 translation modified (item 76)	0.75
Judgment	3.35 (0.78)	-0.22	1 translation modified (item 59)	0.68
Love of learning	4.56 (0.63)	-1.64	-	0.86
Perspective	3.81 (0.80)	-0.92	-	0.81
Bravery	3.70 (0.76)	-0.37	-	0.72
Perseverance	3.74 (0.97)	-0.64	1 item swapped (item 65)	0.85
Honesty	4.29 (0.71)	-1.31	-	0.84
Zest	3.21 (0.99)	-0.31	-	0.84
Love	3.73 (1.05)	-0.57	-	0.87
Kindness	4.23 (0.59)	-0.50	2 items swapped (items 12 and 84)	0.62
Social intelligence	3.95 (0.81)	-0.71	-	0.77
Teamwork	3.25 (1.00)	-0.26	1 item swapped (item 23)	0.80
Fairness	3.81 (0.84)	-0.46	-	0.80
Leadership	3.35 (1.02)	-0.20	-	0.81
Forgiveness	3.16 (0.83)	0.08	-	0.67
Humility	3.32 (0.85)	-0.21	1 item swapped (item 64) 1 translation modified (item 40)	0.71
Prudence	3.57 (0.85)	-0.54	-	0.77
Self-regulation	3.42 (0.81)	-0.42	2 translations modified (items 20 and 92)	0.69
Appreciation of beauty	4.10 (0.76)	-0.87	-	0.67
Gratitude	3.81 (0.78)	-0.46	2 items swapped (items 7 and 55)	0.69
Hope	3.96 (0.88)	-0.77	-	0.77
Humor	3.46 (0.87)	-0.29	-	0.74
Spirituality	3.43 (1.12)	-0.45	-	0.83

Notes.^*^p <.05;^**^p <.01;^***^p <.001 Item references are for the original VIA-IS-M version

### Exploratory factor analysis

The Kaiser-Meyer-Olkin measure of sampling adequacy (MSA) has a value of 0.83 indicating that the data are suitable for factor analysis. Based on parallel analysis, the ideal number of factors is six, while Velicer’s MAP criterion reached its minimum value for three factors. In the analysis, we examined the three-factor solution in more detail (see Table 5).

The three factors collectively account for approximately 41% of the variance in the variables. There are weak to moderate correlations between the factors. The obtained factor structure mostly corresponds to the empirically derived virtues model proposed by McGrath [27]– the 1st factor represents Inquisitiveness, the 2nd factor represents Self-control, and the 3rd factor corresponds to the Caring dimension. However, for particular character strengths, the factor loadings were relatively low (Humility, Hope, Humor, and Spirituality), and some strengths had higher factor loadings in multiple factors (e.g., Perseverance, Perspective, Zest, Fairness, and Hope).

**Table 5:** Results of three-factor solution exploratory factor analyses across three of the studies

	Study 2				Study 3				Study 4		
Strength	1	2	3	1		2	3		1	2	3
Creativity	**0.77**	− 0.05	− 0.09	0.**83**		− 0.14	− 0.03		0.**88**	− 0.07	− 0.15
Curiosity	**0**.**66**	− 0.16	0.13	0.**71**		− 0.04	− 0.01		0.**79**	− 0.09	− 0.03
Judgment	0.00	**0.80**	− 0.28	− 0.01		**0.85**	− 0.25		− 0.12	0.**67**	− 0.02
Love of learning	0.**51**	− 0.08	0.10	0.**46**		0.00	0.09		0.**67**	0.01	− 0.14
Perspective	0.**53**	0.31	0.03	0.**51**		0.29	− 0.04		0.**67**	0.30	− 0.14
Bravery	0.**68**	0.14	− 0.02	.**77**		0.02	− 0.06		0.**72**	0.01	− 0.06
Perseverance	**0.41**	**0.53**	− 0.01	0.14		0.**52**	0.09		0.34	0.**45**	0.10
Honesty	0.04	0.**54**	0.25	0.08		**0.58**	0.02		0.19	0.**45**	0.14
Zest	0.36	0.05	0.**40**	0.25		0.01	0.37		0.26	0.08	0.**48**
Love	0.14	0.01	0.**51**	0.07		− 0.03	0.**54**		− 0.14	0.06	0.**73**
Kindness	0.11	0.03	0.**54**	0.15		0.07	0.**55**		0.27	0.15	0.24
Social intelligence	0.22	0.06	0.**47**	0.29		0.10	0.29		0.**45**	0.10	0.17
Teamwork	− 0.03	− 0.19	0.**42**	− 0.02		− 0.10	0.**45**		0.10	− 0.08	0.38
Fairness	− 0.24	0.35	0.**51**	0.02		0.35	0.37		− 0.08	0.39	0.38
Leadership	0.**62**	0.10	0.02	**0.77**		− 0.05	− 0.09		0.**70**	0.00	0.01
Forgiveness	− 0.20	0.09	0.**49**	0.00		0.04	0.**50**		− 0.24	0.13	0.**58**
Humility	− 0.35	0.22	0.17	**− 0.45**		**0.41**	0.17		− 0.23	**0.44**	− 0.15
Prudence	-0.09	0.**85**	− 0.07	− 0.05		0.**92**	− 0.15		0.03	0.**76**	− 0.03
Self-regulation	0.20	0.**80**	− 0.14	− 0.05		0.**97**	− 0.15		0.22	0.**74**	− 0.03
Appreciation of beauty	0.11	− 0.24	0.**47**	0.07		− 0.13	0.**46**		0.06	− 0.11	0.**42**
Gratitude	0.06	0.05	0.**73**	− 0.07		0.00	0.**81**		− 0.14	0.07	0.**81**
Hope	0.39	0.13	0.37	0.24		0.13	0.39		0.31	0.04	0.**50**
Humor	0.28	0.07	0.16	0.26		0.07	0.14		0.36	− 0.08	0.15
Spirituality	0.06	− 0.04	0.32	− 0.20		− 0.11	0.**56**		0.10	− 0.15	0.32
**Explained variance**	0.15	0.13	0.13	0.14		0.14	0.12		0.18	0.11	
**Factor correlations**											
1	-			-					-		
2	0.17	-		0.35		-			0.21	-	
3	0.47	0.38	-	0.55		0.47	-		0.64	0.29	-
**Correspondence between factor solutions**											
Study 2	-										
Study 3	0.88 (0.72)			-							
Study 4	0.75 (0.42)			0.84 (0.63)					-		
**Factor congruence**											
Study 2	-										
Study 3	0.94, 0.98, 0.96			-							
Study 4	0.95, 0.96, 0.89			0.93, 0.98, 0.91					-		

*Notes*. Factor loadings above 0.40 are in bold; correspondence between factor solutions is measured by the Rand-index, values in parentheses are adjusted Rand-indices; comma-separated list of Tucker’s φ indices of factor congruence are shown for 1st, 2nd and 3rd factor respectively

## Discussion

One of the primary objectives of Study 2 was to address the inadequate reliability observed in specific scales during Study 1 (Gratitude, Humility, and Judgment) and to work toward developing a finalized version of the VIA-IS-M-H. Through targeted revisions of translation and adding new items from the VIA-IS-R item pool, these scales demonstrated improved reliability, all reaching or exceeding the acceptable threshold of α > 0.60. We modified the translation of 2 items from the Self-regulation scale as we found that they were harder to interpret in Hungarian. Notably, the low-reliability scales in the first study—Gratitude, Judgment, and Humility—showed marked improvement. The modifications were informed by item-rest correlations and α-based item deletion criteria. As a result of the changes, a proposed final version of the questionnaire has been developed.

The descriptive statistics revealed a similar pattern to that found in Study 1, with Love of Learning, Curiosity, and Honesty again achieving the highest average scores, yet these scales also displayed significant negative skewness, indicating a ceiling effect. Exploratory factor analysis indicated that a three-factor solution best fits the data, aligning closely with the model proposed by McGrath [26]. Still, some character strengths such as Humility, Hope, Humor, and Spirituality showed relatively weak factor loadings and did not align with one of the factors.

In summary, Study 2 demonstrated that the modifications improved the internal consistency of VIA-IS-M-H. However, further testing is needed to consider this modified version as final, which will be addressed in Studies 2 and 3.

## Study 3

### Method

#### Participants

A total of 264 primary and secondary school teachers participated in the study, recruited through online social media platforms. Participation was voluntary and anonymous, and no compensation was provided. The requirement for participation was that the respondents be actively engaged in teaching work at some educational institution.

Participants (*n* = 41) who potentially produced inconsistent responses were excluded from the analysis, resulting in a final sample of 223 respondents suitable for analysis. Most participants were female (92.4%, *n* = 206), in a relationship (79.8%, *n* = 178), and without exception, possessed a higher education degree. The average age was 47.5 years (SD = 9.32, range: 24–71 years).

### Procedure

The survey was published on the Google Forms platform. Interested individuals could access and complete it through the link posted on the social media platform.

The survey included the final proposed version of the VIA-IS-M-H, followed by the work-related PERMA questionnaire, and two additional questionnaires not used in the current analysis, basic demographic questions (gender, age, relationship status, highest educational attainment), and questions related to the participants’ teaching career (e.g., years of teaching experience, subjects taught, etc.) which were not used in the current analysis.

### Results

#### Descriptive analysis

The highest average scores were observed for the strengths of Love of learning (M = 4.55), Honesty (M = 4.54), and Curiosity (M = 4.40). However, several other strengths scored above 4 (Perseverance, Love, Kindness, Fairness, Appreciation of beauty, Gratitude). Skewness values exceeding 1 were observed for three strengths (Love of learning, Curiosity, Honesty), indicating a slight shift towards higher scores (see Table 6).

**Table 6:** Descriptive statistics from study 3 (incl. Cronbach’s α of strengths, and pearson correlation coefficients with criterion variables)

Strength	M (SD)	Skewness	α	*P*	E	*R*	M	A	Well-being
Creativity	3.93 (0.70)	-0.46	0.79	0.10	0.19	− 0.02	0.21	0.30^**^	0.15^*^
Curiosity	4.40 (0.56)	-1.19	0.76	0.11	0.25^*^	0.07	0.26^*^	0.32^***^	0.22^**^
Judgment	3.47 (0.76)	-0.39	0.72	0.14	0.12	0.07	0.11	0.28^**^	0.17^*^
Love of learning	4.55 (0.61)	-1.55	0.84	0.03	0.21	− 0.04	0.30^**^	0.34^***^	0.15^*^
Perspective	3.85 (0.67)	-0.73	0.76	0.12	0.16	0.10	0.20	0.34^***^	0.19^**^
Bravery	3.67 (0.79)	-0.31	0.77	0.24	0.21	0.05	0.23	0.42^***^	0.26^***^
Perseverance	4.13 (0.72)	-0.73	0.74	0.12	0.20	− 0.05	0.27^*^	0.44^***^	0.21^**^
Honesty	4.54 (0.49)	-1.04	0.75	0.05	0.10	− 0.01	0.19	0.32^***^	0.12
Zest	3.55 (0.84)	-0.46	0.77	0.62^***^	0.57^***^	0.29^**^	0.52^***^	0.50^***^	0.62^***^
Love	4.09 (0.83)	-0.74	0.84	0.33^***^	0.28^**^	0.32^***^	0.32^***^	0.34^***^	0.40^***^
Kindness	4.26 (0.62)	-0.87	0.70	0.28^**^	0.30^**^	0.17	0.34^***^	0.31^***^	0.34^***^
Social intelligence	3.84 (0.71)	-0.57	0.71	0.19	0.19	0.27^**^	0.21	0.20	0.27^***^
Teamwork	3.46 (0.90)	-0.35	0.87	0.24	0.16	0.35^***^	0.21	0.07	0.28^***^
Fairness	4.10 (0.69)	-0.42	0.76	0.23	0.27^*^	0.17	0.32^***^	0.27^*^	0.30^***^
Leadership	3.44 (0.81)	-0.31	0.70	0.25^*^	0.26^*^	0.17	0.2	0.35^***^	0.30^***^
Forgiveness	3.63 (0.83)	-0.51	0.71	0.43^***^	0.25^*^	0.39^***^	0.32^***^	0.18	0.42^***^
Humility	3.19 (0.81)	-0.08	0.73	− 0.09	− 0.07	− 0.12	− 0.01	− 0.06	− 0.09
Prudence	3.53 (0.84)	-0.29	0.82	0.20	0.15	0.14	0.16	0.29^**^	0.23^***^
Self-regulation	3.61 (0.73)	-0.62	0.69	0.20	0.13	0.10	0.18	0.30^**^	0.22^**^
Appreciation of beauty	4.12 (0.67)	-0.59	0.61	0.12	0.27^*^	0.08	0.29^**^	0.23	0.22^**^
Gratitude	4.05 (0.66)	-0.51	0.62	0.31^**^	0.31^***^	0.17	0.37^***^	0.33^***^	0.35^***^
Hope	4.05 (0.78)	-0.92	0.78	0.73^***^	0.56^***^	0.38^***^	0.46^***^	0.56^***^	0.69^***^
Humor	3.65 (0.83)	-0.33	0.76	0.31^***^	0.17	0.25^*^	0.16	0.24	0.29^***^
Spirituality	3.26 (1.19)	-0.18	0.84	0.10	0.04	0.00	0.07	0.08	0.06

Notes. ^*^p <.05; ^**^p <.01; ^***^p <.001

### Reliability

The subscales of the Workplace PERMA questionnaire have shown reliable results for Positive emotions (α = 0.84), Engagement (α = 0.87), Relationships (α = 0.92), Meaning (α = 0.78), Accomplishments (α = 0.78), and Negative aspects of work (α = 0.70).

For the character strengths measured by VIA-IS-M-H, Cronbach’s α values are greater than 0.6 in all cases, indicating acceptable reliability (see Table 6).

### Validity

The workplace well-being index was constructed using principal component analysis based on the six dimensions of the Workplace PERMA questionnaire. The first principal component accounted for approximately 64.1% of the variance in the original scales. The component loadings for Positive emotions, Engagement, Relationships, Meaning, Accomplishments, and Negative aspects of work were 0.54, 0.49, 0.45, 0.29, 0.26, and − 0.35, respectively, indicating that higher values on the first principal component score corresponded to a higher level of well-being.

Character strengths typically showed weak to moderate correlations and, in some cases, relatively strong correlations with well-being and burnout (see Table 6); the associations were positively directed toward well-being and negatively directed toward burnout, consistent with expectations. However, we observed weaker relationships between character strengths and dimensions of well-being, with some strengths only related to Accomplishment (e.g. Judgement, Honesty, Perspective, Creativity) or not related to PERMA dimensions (Spirituality, Humility).

Based on the multiple linear regression analysis results, character strengths significantly explained the variance in well-being with the model explanatory power of 59%. Significant effects were represented by Hope, Zest, Forgiveness, and Spirituality (see Table 3).

Multicollinearity in the model was moderate, with variance inflation factors ranging from 1.23 to 3.62 for the character strengths.

### Exploratory factor analysis

The Kaiser-Meyer-Olkin measure of sampling adequacy (MSA) was 0.83, indicating that the data were suitable for factor analysis. Parallel analysis suggested an ideal number of five factors, while Velicer’s MAP criterion reached its minimum value for three factors. Contrary to the recommended factor numbers, we further examined the three-factor structure to compare our results with those obtained in the second study and the factor structure proposed by McGrath [27].

The three factors collectively accounted for approximately 40% of the variance in the variables (see Table 4). There were moderate correlations among the factors. The obtained factor structure largely corresponds to the empirically derived virtues model proposed by McGrath [27] - the 1st factor represents Inquisitiveness, the 2nd factor represents Self-control, and the 3rd factor corresponds to the Caring dimension. However, for specific character strengths, the factor loadings were relatively low (Zest, Social intelligence, Fairness, Hope, and Humor), while some strengths had higher factor loadings in multiple factors (e.g., Social intelligence, Fairness).

## Discussion

The third study aimed to test the psychometric properties of the proposed final version of the VIA-IS-M-H, focusing on its internal consistency, criterion validity, and factor structure. The scale’s internal consistency was confirmed, as all subscales exceeded the generally accepted threshold of α = 0.60. While most strengths demonstrated satisfactory reliability, Gratitude (α = 0.62) and Appreciation of Beauty and Excellence (α = 0.61) exhibited relatively lower α values.

Regarding criterion validity, the character strengths typically showed weak to moderate correlations with well-being and burnout, consistent with theoretical expectations. Notably, Zest exhibited the strongest and most consistent associations across multiple dimensions of well-being, underscoring its central role in workplace well-being within the teaching profession. In contrast, Spirituality and Humility were unrelated to any well-being dimension, suggesting a limited role in the context of teacher workplace functioning. Interestingly, Creativity, Honesty, and Judgment were specifically linked only to the Accomplishment component of workplace well-being, indicating their more targeted relevance for performance-related outcomes.

The exploratory factor analysis supported a three-factor structure, mostly aligning with the model proposed by McGrath [27], similar to the previous study. However, several strengths—such as Zest, Social Intelligence, Fairness, Hope, and Humor—showed relatively low or diffuse factor loadings.

Although the VIA-IS-M-H demonstrated robust psychometric properties in this teacher sample, the limited generalizability of these results must be acknowledged. The sample consisted exclusively of educators, the majority of whom were female and highly educated. As such, further validation on a more diverse and representative population is necessary to confirm the scale’s broader applicability. This objective was addressed in Study 4.

## Study 4

### Method

#### Participants

478 individuals participated in the study, recruited through online social platforms. Participation was voluntary and anonymous, and no compensation was provided. The only requirement for participation was that the respondents be 18 years of age or older.

We excluded two individuals under 18 years and an additional 84 due to inconsistent responses. Thus, the final sample consisted of 392 participants. The majority of participants were female (81.4%, *n* = 319), had completed or were currently pursuing higher education (75.3%, *n* = 295), and were in a relationship (65.6%, *n* = 257). The average age in the sample was 40.1 (SD = 15.15, range: 18–79 years).

### Procedure

The questionnaire was published on the Google Forms platform. Interested individuals could access and complete it through the link posted on the social media platform.

The battery included the recommended final version of the VIA-IS-M-H, followed by the official Hungarian version of the PERMA-Profiler questionnaire, the SWLS-H measuring life satisfaction, the MET questionnaire measuring mental health, and basic demographic questions (gender, age, relationship status, highest level of education).

### Results

#### Descriptive analysis

The highest average scores were found for Love of learning (M = 4.39), Honesty (M = 4.33), Curiosity (M = 4.28), and Kindness (M = 4.14). For three traits (Honesty, Curiosity, and Love of learning), the skewness index indicates a ceiling effect, as the distribution of scores shifted towards higher scores (see Table 7).

**Table 7:** Descriptive statistics from study 4 (incl. Cronbach’s α for strengths and pearson correlation coefficients with criterion variables)

Strength	M (SD)	Skewness	α	*P*	E	*R*	M	A	Well-being	SWLS-H	Mental health
Creativity	3.89 (0.82)	-0.50	0.81	0.34^***^	0.28^***^	0.13	0.34^***^	0.35^***^	0.34^***^	0.24^***^	0.39^***^
Curiosity	4.28 (0.73)	-1.16	0.80	0.42^***^	0.37^***^	0.17	0.40^***^	0.39^***^	0.41^***^	0.30^***^	0.48^***^
Judgment	3.22 (0.83)	0.01	0.62	0.07	− 0.11	0.08	0.06	0.18	0.08	0.13^**^	0.13^**^
Love of learning	4.39 (0.69)	-1.15	0.83	0.29^***^	0.31^***^	0.08	0.29^***^	0.33^***^	0.29^***^	0.15^**^	0.31^***^
Perspective	3.85 (0.73)	-0.53	0.78	0.35^***^	0.14	0.23^**^	0.36^***^	0.40^***^	0.37^***^	0.29^***^	0.41^***^
Bravery	3.68 (0.83)	-0.39	0.76	0.43^***^	0.19^*^	0.19^*^	0.38^***^	0.37^***^	0.40^***^	0.34^***^	0.47^***^
Perseverance	3.92 (0.85)	-0.75	0.80	0.36^***^	0.07	0.21^**^	0.38^***^	0.58^***^	0.40^***^	0.33^***^	0.40^***^
Honesty	4.33 (0.68)	-1.42	0.81	0.27^***^	0.06	0.18	0.27^***^	0.46^***^	0.30^***^	0.25^***^	0.30^***^
Zest	3.28 (0.97)	-0.34	0.79	0.62^***^	0.30^***^	0.41^***^	0.56^***^	0.53^***^	0.62^***^	0.48^***^	0.57^***^
Love	3.83 (1.03)	-0.59	0.89	0.45^***^	0.14	0.44^***^	0.40^***^	0.31^***^	0.47^***^	0.35^***^	0.44^***^
Kindness	4.14 (0.63)	-0.74	0.66	0.23^**^	0.13	0.13	0.22^**^	0.26^***^	0.24^***^	0.15^**^	0.26^***^
Social intelligence	3.92 (0.78)	-0.77	0.76	0.35^***^	0.11	0.28^***^	0.35^***^	0.31^***^	0.38^***^	0.28^***^	0.44^***^
Teamwork	3.11 (0.99)	-0.18	0.86	0.26^***^	0.16	0.20^*^	0.18	0.13	0.24^***^	0.18^***^	0.28^***^
Fairness	3.73 (0.89)	-0.62	0.82	0.28^***^	0.12	0.12	0.24^***^	0.28^***^	0.26^***^	0.21^***^	0.34^***^
Leadership	3.33 (0.99)	-0.18	0.79	0.42^***^	0.20^**^	0.28^***^	0.39^***^	0.41^***^	0.43^***^	0.36^***^	0.43^***^
Forgiveness	3.22 (0.87)	-0.08	0.69	0.38^***^	0.17	0.25^***^	0.29^***^	0.23^***^	0.35^***^	0.33^***^	0.41^***^
Humility	3.23 (0.87)	-0.11	0.72	− 0.15	− 0.21^**^	− 0.19^*^	− 0.17	− 0.07	− 0.20^***^	− 0.14^**^	− 0.14^**^
Prudence	3.53 (0.84)	-0.25	0.78	0.15	− 0.08	0.13	0.17	0.32^***^	0.18^***^	0.19^***^	0.22^***^
Self-regulation	3.50 (0.77)	-0.44	0.66	0.32^***^	− 0.04	0.22^**^	0.34^***^	0.51^***^	0.35^***^	0.30^***^	0.35^***^
Appreciation of beauty	3.93 (0.77)	-0.89	0.69	0.20^*^	0.27^***^	0.18	0.23^***^	0.14	0.24^***^	0.15^**^	0.24^***^
Gratitude	3.85 (0.77)	-0.54	0.65	0.43^***^	0.19^*^	0.32^***^	0.40^***^	0.31^***^	0.44^***^	0.32^***^	0.41^***^
Hope	3.95 (0.89)	-0.81	0.79	0.81^***^	0.28^***^	0.47^***^	0.74^***^	0.64^***^	0.78^***^	0.63^***^	0.75^***^
Humor	3.53 (0.91)	-0.31	0.76	0.41^***^	0.16	0.24^***^	0.25^***^	0.24^***^	0.34^***^	0.25^***^	0.48^***^
Spirituality	3.22 (1.16)	-0.25	0.84	0.23^***^	0.21^**^	0.10	0.24^***^	0.18^*^	0.23^***^	0.24^***^	0.20^***^

*Notes*. ^*^p <.05; ^**^p <.01; ^***^p <.001

### Reliability

The reliability was found to be acceptable in the SWLS-H (α = 0.89), the Mental Health Test Well-being (α = 0.91), Savoring (α = 0.76), Creative and executive social efficiency (α = 0.76), Self-regulation (α = 0.71), Resilience (α = 0.85), PERMA-Profiler Positive emotions (α = 0.89), Relationship (α = 0.80), Meaning (α = 0.88), and Accomplishments (α = 0.73) scales. In contrast the Engagement scale did not reach an acceptable level of reliability (α = 0.52).

For each character strength in the VIA-IS-M-H, we found an acceptable level of reliability (see Table 7).

### Validity

The well-being measure, formed based on the five dimensions of the PERMA-Profiler questionnaire using principal component analysis, retained approximately 65.6% of the total variance of the scales. The loadings associated with the first principal component for the Positive emotions, Engagement, Relationship, Meaning, and Accomplishments scales were 0.54, 0.20, 0.50, 0.57, and 0.30, respectively. Accordingly, a high score on the first principal component indicates a higher level of well-being.

In the case of the Mental Health Test, the first principal component retained approximately 50.9% of the total variance of the five scales. The weights for the Well-being, Savoring, Creative and executive social efficiency, Self-regulation, and Resilience scales in the first principal component were 0.57, 0.40, 0.29, 0.32, and 0.57, respectively. Thus, a high score on the first principal component reflects a higher level of mental health.

The character strengths generally showed weak to moderate correlations with well-being, life satisfaction, and mental health, aligning with the expected directions (see Table 7). The correlation patterns of character strengths with the dimensions of well-being were similar to those observed in the first study. Most correlations were weak to moderately positive, except Humility, which showed slight negative correlations, and Judgment, which had no significant relationships with any dimension of PERMA.

Based on the results of multiple linear regression analyses, the character strengths demonstrated sufficient predictive power for well-being (R^2^ = 0.66), life satisfaction (R^2^ = 0.46), and mental health (R^2^ = 0.65). Hope and Zest showed significant effects in all three models. At the same time, Love and Self-regulation had a significant impact only on well-being, and Humor and Bravery had significant effects only on mental health. Forgiveness and Curiosity had significant effects on both mental health and life satisfaction (see Table 3).

The models showed a moderate level of multicollinearity, with variance inflation factors ranging between 1.18 and 3.04.

### Exploratory factor analysis

The Kaiser-Meyer-Olkin measure of sampling adequacy (MSA) was 0.87, indicating that the data is suitable for factor analysis. According to parallel analysis, the ideal number of factors is seven, while the minimum value of Velicer’s MAP criterion was reached for three factors. Similar to previous studies, we further examined the three-factor solution.

The three factors collectively explain approximately 39% of the variance in the variables. There are moderate correlations among the factors. The obtained factor structure mostly aligns with the empirically derived virtues model proposed by McGrath [27] - Factor 1 corresponds to Inquisitiveness, Factor 2 to Caring, and Factor 3 to Self-control. The factor loadings were relatively low for several strengths (Judgment, Kindness, Teamwork, Humor, Spirituality).

## Discussion

The primary aim of Study 4 was to further validate the final version of the VIA-IS-M-H in a broader, more heterogeneous adult sample and assess its psychometric properties with respect to internal consistency, criterion validity, and factor structure. The findings support the instrument’s robustness, building on previous studies’ results. Internal consistency was acceptable across all 24 character strength subscales, with Cronbach’s α values mostly exceeding the 0.70 threshold. A few strengths, such as Gratitude, Kindness, Appreciation of Beauty, and Self-regulation, had lower reliability coefficients (0.65–0.69), which may benefit from item refinement in future revisions.

Character strengths displayed significant, moderate correlations with well-being, life satisfaction, and mental health. Hope and Zest consistently emerged as strong predictors of all the measured variables. The correlation pattern of the character strengths with the well-being dimensions was consistent with the first study’s findings. Judgment showed no significant correlation with any well-being dimensions, and Humility was negatively associated with multiple outcomes, which aligns with our previous results and Wagner’s results [43]. It should be noted that the Engagement scale of the PERMA Profiler demonstrated low internal consistency (α = 0.52), warranting caution in interpreting these results.

Exploratory factor analysis aligns with the previous results, suggesting the three-factor solutions, yet some character strengths (Judgment, Kindness, Teamwork, Humor, and Spirituality) displayed weak or diffuse loading.

### Similarity of factor solutions

In analysing the agreement between factor structures found in the second, third, and fourth studies, we calculated Rand indices and adjusted Rand indices. The high values of these indices (ranging from 0.75 to 0.88) indicate high similarity between the factor structures. In contrast, the non-zero values of the adjusted indices (ranging from 0.42 to 0.72) suggest a degree of agreement between the factor structures that exceed the level of pure chance agreement (see Table 5).

According to Tucker’s φ measure of factor congruence we found fair to good congruence between the factor solutions for each pair of studies with φ values between 0.94 and 0.98 for the second and third studies, between 0.89 and 0.96 for the second and fourth studies, and between 0.91 and 0.98 for the third and fourth studies (see Table 5).

Table 8 presents the categorization of VIA character strengths based on exploratory factor analyses conducted in Studies 2, 3, and 4 and compares them to McGrath’s [26] theoretical classification. The results show a generally stable three-factor solution across the three studies, with most strengths consistently aligning with one of the derived factors. Character strengths classified by McGrath [26] under Inquisitiveness (e.g., creativity, curiosity, love of learning, perspective, bravery) consistently loaded onto the same factor across all three studies. Similarly, strengths associated with Self-control in McGrath’s model [26], like prudence and self-regulation, consistently loaded on the Self-control factor across all studies. Caring strengths (e.g., love, forgiveness, gratitude) also showed stable loadings.

**Table 8:** Classification of character strengths based on our studies with the proposed classification of McGrath [26] side by side demonstrating the degree of overlap between them

	Our classification			
Strength	Study 2	Study 3	Study 4	McGrath’s [26] classification
Creativity	inquisitiveness	inquisitiveness	inquisitiveness	inquisitiveness
Curiosity	inquisitiveness	inquisitiveness	inquisitiveness	inquisitiveness
Judgment	self-control	self-control	self-control	inquisitiveness, self-control
Love of learning	inquisitiveness	inquisitiveness	inquisitiveness	inquisitiveness
Perspective	inquisitiveness	inquisitiveness	inquisitiveness	inquisitiveness, self-control
Bravery	inquisitiveness	inquisitiveness	inquisitiveness	inquisitiveness
Perseverance	inquisitiveness, self-control	inquisitiveness	inquisitiveness	self-control
Honesty	self-control	inquisitiveness	self-control	self-control
Zest	caring	-	caring	inquisitiveness
Love	caring	caring	caring	caring
Kindness	caring	caring	-	caring
Social intelligence	caring	-	inquisitiveness	inquisitiveness
Teamwork	caring	caring	-	caring
Fairness	caring	-	-	self-control, caring
Leadership	inquisitiveness	inquisitiveness	inquisitiveness	caring
Forgiveness	caring	caring	caring	caring
Humility	-	inquisitiveness, self-control	self-control	self-control
Prudence	self-control	self-control	self-control	self-control
Self-regulation	self-control	self-control	self-control	self-control
Appreciation of beauty	caring	caring	caring	inquisitiveness, caring
Gratitude	caring	caring	caring	caring
Hope	-	-	caring	inquisitiveness
Humor	-	-	-	inquisitiveness, caring
Spirituality	-	caring	-	caring

*Notes.* Classification of strengths based on our factor analysis results (only shown where factor loading > 0.4 in at least one factor). Category labels are adapted from McGrath [26] based on degree of overlap with our categorisation

Despite the overall agreement, several character strengths exhibited notable deviations from McGrath’s [26] empirically derived virtues model. Mcgrath [26] classified perseverance under self-control, but our data is loaded on both Inquisitiveness and self-control in Study 2 and solely on Inquisitiveness in Studies 3 and 4. Honesty also demonstrated inconsistent placement, appearing under Self-control in Studies 2 and 4 but under Inquisitiveness in Study 3. Likewise, humility was only retained in Studies 3 and 4, with factor loadings pointing to Self-control, consistent with McGrath’s [26] model. Zest, which McGrath [26] links to Inquisitiveness, appeared under caring in Studies 2 and 4 while did not reach the loading threshold in Study 3. Hope, humor, and spirituality showed weak or no loadings above the 0.40 threshold in at least two studies, suggesting less consistent integration into the derived factor structure. McGrath [26] assigns all three to either Inquisitiveness or caring, yet they did not robustly emerge in our data.

Overall, our findings regarding the correspondence between factor structures and the similarity of factor solutions on the level of factor loadings support the stability of the suggested structure of strengths proposed by McGrath [26].

### General discussion

In addition to its widespread usage, McGrath [29] highlighted that the VIA-IS questionnaire has several limitations and undertook a comprehensive revision process, resulting in the VIA-IS-R questionnaire package. Our study aimed to validate the Hungarian version of the VIA-IS-M questionnaire. Notably, the revised version of the VIA-IS questionnaire has not previously been used on a Hungarian sample, underscoring the novelty and significance of the study.

The VIA-IS-M-H questionnaire was tested in 4 studies on different samples, with 1878 participants. Descriptive statistics showed similarities across the samples regarding mean scores for the strengths; Love of Learning, Curiosity and Honesty all had high mean scores, consistent with the results of McGrath and Wallace [30]. Spirituality had low scores in all samples (M = 3.26–3.43); Forgiveness also had low mean scores (M = 3.16–3.63). For the Curiosity, Love of Learning and Honesty scales, extreme distributions of scores (skewness) were found in all four studies. Character strengths are socially desirable, which may explain the bias towards higher scores.

In the first study, we found markedly low internal consistency for Gratitude, Humility and Judgment scales (α < 0.6) and relatively low internal consistency for Kindness, Self-regulation, and Appreciation of beauty and excellence scales (α < 0.7). Based on these results, we aimed to improve the internal consistency of the scales in Study 2. The translation modifications and new items in the scales improved reliability, with all subscales reaching the minimum reliability value. The translation modification benefited the Self-regulation scale. By changing two items, the scale could be interpreted more easily in Hungarian. However, the internal consistency of the Kindness scale (α = 0.62) remained relatively low. Using the final version of the VIA-IS-M-H in Study 3 and Study 4, we found low reliability for Gratitude (α = 0.62) and Appreciation of beauty and excellence (α = 0.61) in Study 3 and low reliability for Judgement (α = 0.62), Gratitude (α = 0.65) and Kindness (α = 0.66) in Study 4. These results are consistent with previous studies using the VIA-IS-R [30, 31], where the VIA-IS-M format had the lowest internal reliability among the revised VIA instruments. Like our findings, McGrath et al. [31] reported relatively low but acceptable reliability for Gratitude, Appreciation of Beauty and Excellence, Judgment, and Humility. These lower reliability coefficients may be attributed to the reduced number of items in the VIA-IS-M format and the more complex interpretation of the reverse items compared to the VIA-IS-P. To conclude, these scales would benefit from an item revision to achieve more stable internal consistency.

We examined correlation coefficients between character strengths and criterion variables to assess the VIA-IS-M-H questionnaire’s criterion validity. There was a weak-to-moderate correlation between character strengths and criterion variables, with the direction of correlations in line with the prior expectations. The strongest correlations with the well-being variables were between the Hope and Vitality character strengths. These strengths, known as the “happiness strengths”, have been shown by several studies to be closely related to well-being [22, 44]. When examining the correlation pattern between character strengths and the PERMA dimensions, weak to moderate positive correlations were found, similar to the findings of Wagner et al. [43]. Notably, Humility showed a small negative relationship with well-being dimensions. Judgement had no significant connections, suggesting that these strengths may not necessarily contribute to a good life; instead, they may help individuals avoid negative experiences [43]. The findings suggest a potential difference in the relationship between character strengths and workplace well-being compared to general well-being. Overall the relationships between character strengths and workplace well-being were weaker. Specific character strengths - Creativity, Bravery, Perspective, Honesty - showed significant relationships only with the Accomplishment dimension of workplace well-being, whereas, for general well-being, relationships were observed with the other well-being dimensions. However, these results should be interpreted cautiously, as the second study was conducted with a small, specific sample of teachers, and more research is needed to draw long-term conclusions.

We conducted multiple linear regression analyses to determine how character strengths can predict criterion variables. We used character strengths as predictor variables in the regression models and controlled for age and gender. The character strengths demonstrated sufficient predictive power for all the criterion variables in all studies (R^2^ = 0.43-0.71), and the highest explained variance was for well-being (R^2^ = 0.71). Hope and Zest showed the highest effect in almost all models.

We examined the higher-order organisation of character strengths using exploratory factor analysis. Rand indices were used to analyse the agreement between factor structures found in the second, third, and fourth studies. Degree of agreement between the factor structures that exceed pure chance agreement. According to Tucker’s φ measure of factor congruence we found fair to good congruence between the factor solutions in the studies. A three-factor solution was tested and the results are consistent with recent studies on the factor structure of character strengths [27, 28]. The data suggest that our three-factor model shows a high degree of similarity with the factors proposed by McGrath et al. [27] (Caring, Inquisitiveness, and Self-control). Yet, our data do not show a complete fit, some strengths had higher factor loadings in multiple factors (e.g., Social intelligence, Fairness) and several character strengths did not load consistently across studies or failed to reach the threshold for inclusion in a specific factor (e.g., Hope, Humor, Spirituality), suggesting that their higher-order classification may be more context-dependent or less robust. Notably, our results indicate a slightly broader Inquisitiveness factor that includes character strengths such as leadership and perseverance, which are typically categorized into other factors in McGrath’s [26] model. These differences may be attributed to variations in sample composition, language adaptation, or cultural interpretations of specific strengths. Overall, the findings support the suggested structure of strengths proposed by McGrath [28].

The study has several limitations, such as the sample not being representative and the participants being mainly women with higher education degrees. In order to obtain more generalisable data, it would be practical to test the VIA-IS-M-H on a gender-balanced sample and in different occupational groups. In addition, the studies relied on cross-sectional data, so longitudinal data would help compare test-retest reliabilities.

## Conclusion

VIA-IS-M is a shorter form of the VIA-IS-R instruments, which is suitable when seeking a briefer measurement and addressing the response biases inherent in positively formulated items [30]. With the limitations in mind, the results suggest that VIA-IS-M-H demonstrate generally acceptable reliability and validity levels, and it is a suitable instrument for assessing character strengths, confirming the results of previous studies [30, 31].

## Data Availability

The dataset used and analysed in the current study is available from the corresponding author upon reasonable request.

## Abbreviations

MAP: Maximum Average Partial
MET: Mental Health Test
MSA: Measure of Sampling Adequacy
SWLS-H: Hungarian adaptation of the Satisfaction with Life Scale
VIA-IS: VIA Inventory of Strengths
VIA-IS-M: VIA Inventory of Strengths- „Mixed”
VIA-IS-M-H: Hungarian version of VIA Inventory of Strengths- „Mixed”
VIA-IS-P: VIA Inventory of Strengths- „Positive”
VIA-IS-R: VIA Inventory of Strengths-Revised
VIF: Variance Inflation Factor
